# Methodology for a reverse engineering process chain with focus on customized segmentation and iterative closest point algorithms

**DOI:** 10.1016/j.mex.2022.101640

**Published:** 2022-02-26

**Authors:** Stephan Mönchinger, Robert Schröder, Rainer Stark

**Affiliations:** a Fraunhofer IPK, Germany; b Technical University Berlin, Germany

**Keywords:** Cad repositioning, Reverse engineering, Segmentation, ICP, PCL, Open source development

## Abstract

One-off construction is characterized by a multiplicity of manual manufacturing processes whereby it is based on consistent use of digital models. Since the actual state of construction does not match the digital models without manually updating them, the authors propose a method to automatically detect deviations and reposition the model data according to reality. The first essential method is based on the “Segmentation of Unorganized Points and Recognition of Simple Algebraic Surfaces” presented by Marek Vanco (2003). The second method is the customization of the iterative closest point (ICP) algorithm. The authors present the overall structure of the implemented software, based on open source and relate it to the general reverse engineering (RE) framework by Buonamici et al. (2017).

A highlight will be given on.

• The general architecture of the software prototype.
• A customized segmentation and clustering of unorganized points and recognition of simple algebraic surfaces.
• The deviation analysis with a customized iterative closest point (CICP) algorithm.

Especially in the field of one-off construction, characterized by small and medium companies, automated assessment of 3D scan data during the design process is still in its infancy. By using an open source environment progress for consistent use of digital models could be accelerated.

## Graphical abstract

**Figure:**
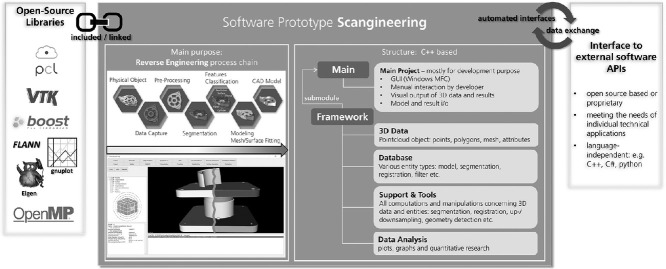

**Specifications table**

**Table:** 

Subject area;	Engineering
More specific subject area;	Virtual Product Creation
Method name;	Development of a software prototype for the RE process chain with focus on customized segmentation and iterative closest point algorithms
Name and reference of original method;	RE process chain: Francesco Buonamici, Monica Carfagni, Rocco Furferi, Lapo Governi, Alessandro Lapini & Yary Volpe 2017. Reverse Engineering modeling methods and tools: A survey. In Computer-Aided Design and Applications. DOI: 10.1080/16,864,360.2017.1397894Segmentation algorithm: Marek Vanco 2003. A direct approach for the segmentation of unorganized points and recognition of simple algebraic surfaces.ICP algorithm: Timo Zinßer, Jochen Schmidt & Heinrich Niemann 2003. A refined ICP algorithm for robust 3-D correspondence estimation. In Proceedings 2003 International Conference on Image Processing. DOI: 10.1109/ICIP.2003.1246775
Resource availability;	PCL [4]: https://pointclouds.org/VTK: https://vtk.org/boost: https://www.boost.org/FLANN: https://github.com/mariusmuja/flannEigen: https://eigen.tuxfamily.org/index.php?title=Main_Pagegnuplot: http://www.gnuplot.info/gnuplot-iostream: http://stahlke.org/dan/gnuplot-iostream/OpenMP: https://www.openmp.org/Windows MFC: https://docs.microsoft.com/en-us/cpp/mfc/mfc-desktop-applications?view=msvc-160git submodule: https://git-scm.com/book/de/v2/Git-Tools-Submodule

**Method details**.

## Background

We want to reposition given CAD models from an assembly (“as-designed”-state) according to underlying 3D scan data (“as-is” state). The use case is an aircraft's interior, where parts like furniture (seats, floor) and other components attached to the aircraft's body are demounted. The scan data is present as tessellated point cloud. It features a relatively bad quality, meaning that the point density is coarse and details smaller than 10 mm are not depicted. Noise artefacts distort the true representation of the geometry. To accomplish this repositioning, we make use of our software framework that is capable of state-of-the-art tools of the RE-process chain (see [1]). In the scope of this paper, we want to describe the development and general architecture of this prototype. The whole code is open source based. To meet the special requirements of the given use case, we customized common methods: First, the segmentation method and second, the iterative closest point method. In supplementary material, we give further information about the automated process presented in our corresponding paper.

## Our framework: Software prototype scangineering

The graphical abstract depicts the architecture and principle of our software prototype. As integrated development environment (IDE), we use Visual Studio Professional 2019. The project is split in main project and framework. The main project is generally for development and testing purposes. It contains a GUI, implemented with *Windows MFC*. The user is able to import and export data and manually call all data-processing actions. Using the visualizer of *PCL*, data is displayed in the main window. The framework is a separate project which is added to the main project as a submodule. It includes all of our functionalities to store, manipulate and analyse 3D scan data. The reason and advantage of separating main project from framework is that it enables to include the framework as “invisible” back-end for further software solutions (e.g. as backend for a plugin in proprietary software). As further benefit, the backend does not need to be compiled each time the GUI is adapted. The framework's subprojects are compiled as static libraries and therefore perform fast when included in the main project. It consists of four C++ subprojects:

• **3D data**: The central object is the so-called “PC_Object” (stemming from point cloud object). It contains all datatypes that characterize a 3D object. This may include: point cloud, polygon mesh, point normal, polygon normal, edge graph, area, volume, centroid, principle axes, etc.
• **Database**: The database inherits all different entities that may be constructed from 3D data. A model itself (mostly representing a single PC_Object), segmentation, registration, correspondences, geometry detection, NURBS surfaces, filter, etc. If any other entity type is needed for individual purposes, this database may be extended easily by adding a corresponding class. A database object that is able to access and query all entities completes this structure.
• **Support & tools**: In this structure, all manipulations to obtain the different entities are carried out. Here, the true “work” is done, whereas the entities in the database only store and organize the outcome of the tools. Similar to the entity types, objects like segmentation or registration exist. Additionally, the static class called “Tools” contains a variety of data processing algorithms, it is used throughout all other objects.
• **Data analysis**: This subproject represents the link to *gnuplot*. Quantitative data is given as input (e.g. the distribution of surface types and sizes of segmentation results) to generate plots and graphs. This is carried out by a scripting interface using simple *gnuplot* commands.

The external libraries that we use are.

• *PCL (Point Cloud Library):* framework that delivers a broad basis for working with point clouds and polygon meshes. It can be installed separately or as an all-in-one installer, that also includes third party libraries *VTK, Boost, Flann* and *Eigen*.
• *VTK (Visualization ToolKit):* Library for 3D computer graphics. The *PCL* visualizer is based on *VTK*. Additionally, it provides useful datatypes for polygon meshes and graph structures.
• *Boost:* support for *PCL* concerning mathematics and datatypes.
• *FLANN (Fast Library for Approximate Nearest Neighbours):* Delivers the Octree and KdTree structures. Also used by *PCL*.
• *Eigen:* Tools and datatypes for mathematics: vectors, matrices, linear algebra, linear solvers.
• *Gnuplot:* For plotting 2- and 3-dimensional graphs. Implemented using the open source *gnuplot-iostream* project (see Resource availability above)
• *OpenMP:* used to parallelize algorithms. Very intuitive and straight forward to implement.

The optional interfaces to external software APIs enable to integrate the *Scangineering* framework in existing software environments. To meet the needs of individual technical applications, diverse implementations are conceivable. Depending on given conditions, it could be either open source based or implemented in a proprietary software, in a suitable programming language. Regarding the given use case as an example, a link to the proprietary CAD software *SolidWorks* is required, which is the desired front-end for the end user. Therefore, we used the API in C#.

## Customized method: Segmentation by Vanco

Marek Vanco [2] presented a segmentation method based on normal vectors that includes a variable reference vector. The aim is to detect planar faces. The algorithm uses the region growing principle, meaning that starting from an initial point, a segment is created by iteratively checking the difference of normal vectors of neighbouring candidates. The variable reference vector is the average normal vector of all points already belonging to the segment. It is updated each time a point is added. Thus, two conditions are imposed: first, a condition for local curvature by checking the candidate's normal vector against the segment's neighbouring normal vector. Second, a condition on global orientation of the candidate's normal vector by checking against the reference vector. We implemented Vanco's algorithm as it is, and additionally we simplified it: We added the option to fix the reference vector, such that segments with a desired orientation are detected. This turned out to be useful in our use case, since in the global CAD-assembly, orientations of surfaces are already known. To detect scan segments that correspond to a certain CAD surface, we simply used the known CAD surface normal vector as reference vector. As initial data, a polygon mesh is preferred for the segmentation. Using a mesh, the information about neighbours are given through edge and vertex connections. This makes a separate neighbour search unnecessary.

In the following, the essential elements of how we implemented the segmentation algorithm of Vanco using the variable reference vector is presented as pseudo code. The algorithm constitutes one region growing cycle, starting with one random point of the point cloud, called “seed”. To obtain our variant using a fixed reference vector, line 6 becomes $v_{ref}=fixed\;vector$ and line 17 is dropped. The algorithm is also simplified to no usage of a reference vector if all three lines 6, 14 and 17 are dropped and only the local condition is checked in line 15, obtaining a more general approach for a segmentation method. All variants have their advantages and disadvantages, depending on the actual application.

Variable names and explanations:

**Table:** 

P	all points	$\alpha_{l}$	local normal vector difference
S	all points in segment	$\alpha_{r}$	global normal vector difference to reference vector
B	all points on segment boundary	$\tau_{l}$	threshold local angle criterion
C[p]	All neighbouring candidates of point p	$\tau_{r}$	threshold global angle criterion to reference vector
$p_{c}$	current point	$v_{ref}$	reference vector
$p_{t}$	test point		

Pseudo code:

**Table:** 

1	// initialization	
2	determineC[p]∀p∈P	// determine all neighbours (candidates)
3	$p_{c}=random\;point\;\in P$	// determine random seed
4	$S.add(p_{c})$	// add seed to segment
5	$B.add(C[p_{c}])$	// add neighbours of seed to boundary points
6	$v_{ref}=normal\;of\;p_{c}$	// determine first reference vector
7	// region growing	
8	while(B≠empty)	// while there are any boundary points
9	$while\;(C[p_{c}]\neq empty)$	// while there are still candidates for current point
10	$p_{t}=C\left[p_{c}\right]\left[0\right]$	// test point
11	$v_{1}=normal\;of\;p_{c}$	
12	$v_{2}=normal\;of\;p_{t}$	
13	$\alpha_{l}=v_{2}∡v_{1}$	
14	$\alpha_{r}=v_{2}∡v_{ref}$	
15	$if\;(\alpha_{l}<\tau_{l}\;AND\;\alpha_{r}<\tau_{r})$	// check for local and global criterion
16	$S.add(p_{t})$	// add test point to segment
17	$update\;v_{ref}\;with\;v_{2}$	// update reference vector by calculating new average Vector
18	$B.add(C[p_{t}])$	// add all candidates of new point to boundary points
19	$C\left[p_{c}\right].erase\left(p_{t}\right)$	// erase test point from candidates of current point
20	$B.erase(p_{c})$	// erase current point from boundary points
21	if(B≠empty)	// if boundary points left, define new current point as one of boundary points
22	$p_{c}=B\left[0\right]$	

To obtain segmentation results for not only one start point but the whole model, many or even all points of the model can be used as start points. Another strategy could be to choose the next seed out of the remaining points after one region growing cycle. If one does not explicitly exclude already in other segments used points, segments may overlap, thus having points in common. Ideally, each point on one segmented surface generates the same surface when used as start point. The fact of having overlapping patches lead to a further customization that we made in the subsequent step after segmentation.

**Fig. 1:**
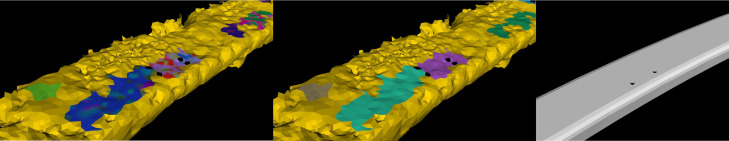
Cluster formation of scan segments. Left: patches without clustering; middle: clustered patches; right: corresponding CAD part.

As mentioned, the scan data in the use case exhibits many noise artefacts and is very coarse at the same time, leading to very rough representations of surfaces. This is harmful for the segmentation results, since the local criterion is hard to fulfil. The angular threshold for the local condition may be increased, but a too large threshold may lead to regions, that erroneously traverse geometry edges. The seeds for the region growing segmentation are spread randomly over the scan data. Results of the segmentations are mostly small separate patches that initially belong to one single continuous surface. To counteract this problem, we invented a strategy to fuse segmentation results to larger clusters. Iteratively, all patches are examined if they overlap others – if so, by a certain minimum number of points, they will be merged Fig. 1. shows the principle. Left, the segmentation results without the cluster formation are depicted. Several small patches are next to and overlap each other, shown in different colours. Middle, the same patches are merged into larger clusters. This leads to a more proper description of the surface, despite of the bad scan data quality. Right, the corresponding CAD part is shown for this scan section, where the continuous planar surface is facing upwards.

## Customized method: Iterative closest point

The Iterative Closest Point (ICP) algorithm is a well-known tool for registration, aiming to minimize the distance between two point clouds by aligning the source point cloud with the target [3]. To this end, for each point in the source cloud, the nearest point in the target is determined and the summed squared distance of all point pairs is calculated. This value needs to be minimized iteratively by finding suitable transformations (rotations and translations) of the source cloud. Since regarding our use case we need to align CAD data with scan data, it seems evident to use this algorithm. *PCL* provides a ready-to-use implementation of ICP.

However, as a project requirement only transformations or rotations in specified degrees of freedom (DoF) were allowed. Therefore, we implemented a customized version and labelled it “Custom ICP” (CICP) that only takes one single DoF for the transformation into account. As measure for the distance between source and target, we took the average over all absolute pairwise distances instead of squared distances and termed it “fitness score”:

$$fitness\;score=\;\frac{\sum_{\;i=0}^{\;n_{points}}\left(\left|\;distance\left[i\right]\;\right|\right)\;}{n_{points}}$$

In the use case, the source is the CAD data and the target the 3D clusters resulting from the aforementioned segmentation. One CICP cycle is solely applied per surface, or rather for all surfaces that have the same orientation. For instance, a surface having a normal vector (1,0,0) is not suited approximate along y or z axes, but only in x direction. The following figure left shows a typical situation: from two surfaces with different orientations, scan sections could be obtained. Separately for each surfaces, the CICP is applied in the respective direction. Right, a close up of the scan sections (of the curved surface, left red framed) are depicted, showing the cad to scan fitting before (top) and after (bottom) the CICP process.

**Fig. 2:**
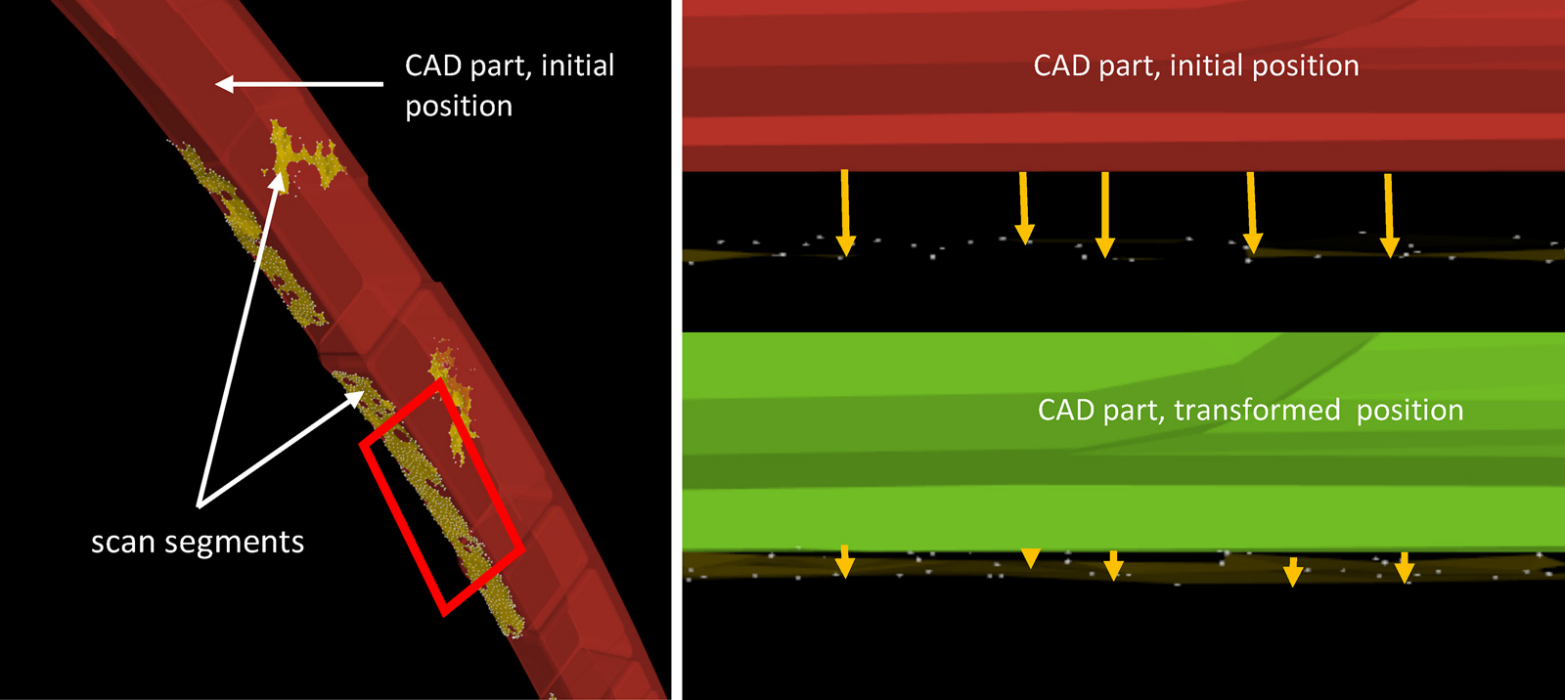
Left: exemplary scan segments and CAD data; Right Top: scan segments and CAD initial position; Right bottom: scan segments and transformed CAD position.

We obtained the source cloud as follows: the CAD data is imported as .stl file, such that we can upsample points on the polygon faces and afterwards downsample these to obtain a homogeneous distributed point cloud having a higher point density than the scan segments. Furthermore, there were several pre-processing steps required, which we described more in detail in our corresponding paper. The scan patches in Fig. 2 are already exempted from erroneous noise and outliers, thus ready for the CICP. The implementation is straightforward and presented as pseudo code in what follows. To obtain a registration that approximates in coarse steps at the beginning and refine steps when getting closer, we introduced certain refinement levels. Finding out which values for $n_{r},$ $x_{r}$ and initial transformation matrices was achieved by trying and testing. A more intelligent solution that automatically determines and adapts to the current situation is conceivable and desirable in future. This CICP cycle depicts the alignment in one DoF. To transform the source along different DoFs, we applied these consecutively and tested different combinations.

Variable names and explanations:

**Table:** 

$P_{s}$	source points	$n_{r}$	number of amplitude refinements; typically $n_{r}<5$
$P_{s,n}$	source points transformed	$f_{c}$	current fitness score
$P_{t}$	target points	$f_{n}$	new fitness score
x	factor for amplitude of transformation	M	4x4 transformation matrix
$x_{r}$	Refinement factor for x, typically 0.01<x<0.25		

Pseudo code:

**Table:** 

1	// initialization	
2	*type= determine type of transformation; could be "translation" or "rotation"*	
3	*axis* = *determine translation or rotation axis; could be* "*x*", "*y*", "*z*", *or* "*abitrary [x, y, z]"*	
4	M=determinetransformationmatrix(type,axis)	// transformation matrix set up according to given type and axis
5	x=1	
6	// iterative transformation	
7	$for\;(i=0;\;0<n_{r};\;i++$)	
8	M.scale(x)	// scale the transformation matrix according to factor
9	$f_{a}=compute\;fitness\;score\left(P_{s},\;P_{t}\right)$	// determine initial fitness score
10	$P_{s,n}=transform\left(P_{s},\;M\right)$	// transform
11	$f_{n}=compute\;fitness\;score\left(P_{s,n},\;P_{t}\right)$	// determine new fitness score
12	$if\;(f_{n}>f_{a})$	// check if first transformation yields improvement; if not, switch direction
13	M=−M	
14	$while\;(f_{n}<f_{a}$)	// do while fitness score improves
15	$f_{a}=f_{n}$	
16	$P_{s}=P_{s,n}$	
17	$P_{s,n}=transform\left(P_{s},\;M\right)$	
18	$f_{n}=compute\;fitness\;score\left(P_{s,n},\;P_{t}\right)$	
19	$x=x*x_{r}$	// refine factor for transformation

Furthermore, we adapted the CICP to perform a parallel alignment. To this end, the fitness score in the algorithm is replaced by the average deviation of all distances to the fitness score:

$$average\;deviation=\;\frac{\sum_{\;i=0}^{\;n_{points}}\left(\left|\;distance\left[i\right]-f_{a}\;\right|\right)\;}{n_{points}}$$

In line 12, the criterion states that the transformation is iteratively repeated until the average deviation is minimized. If two point clouds with similar geometric structure are parallel regardless their absolute distance, the distances of corresponding point pairs are everywhere equal, resulting in the deviation of distances going to zero. This method turned out to be necessary and well performing, since CAD parts could first be aligned parallel and afterwards transformed to minimize the distance to the scan.

## Declaration of Competing Interest

The authors declare that they have no known competing financial interests or personal relationships that could have appeared to influence the work reported in this paper.

## Appendix Supplementary materials

Image, application 1

## References

[1] Buonamici Francesco, Carfagni Monica, Furferi Rocco, Governi Lapo, Lapini Alessandro, Volpe Yary (2017). Reverse engineering modeling methods and tools: a survey. Comput. Aided Des. Appl..

[2] Marek Vanco 2003. A direct approach for the segmentation of unorganized points and recognition of simple algebraic surfaces. 2003. Thesis for: Doctoral Advisor: Guido Brunnett.

[3] Zinßer Timo, Schmidt Jochen, Niemann Heinrich (2003). Proceedings of the International Conference on Image Processing.

[4] Rusu Radu Bogdan, Cousins Steve (2011). Proceedings of the IEEE International Conference on Robotics and Automation (ICRA).

