# Supplementary material for: Methodology for a reverse engineering process chain with focus on customized segmentation and iterative closest point algorithms
Source: MethodsX. 2022 Feb 26;9:101640. doi: 10.1016/j.mex.2022.101640 (PMC8902605; doi:10.1016/j.mex.2022.101640)
Supplement: Supplementary file 1 [file mmc1.docx]

**Supplementary material and additional information:**

*Approach for CAD part characterizations*

In our corresponding paper, in section *3.2 Back-End: Main Process* we described the classification of CAD components. To avoid the characterization of part types being hard-coded, we set up an MS Excel .csv with the relevant characterizations, depicted in Table 1. In this way, other parts could simply be integrated in the repositioning process. Attributes are the global position, number of surfaces that should ideally be identified, face type, a face characterization type and surface area range that additionally enabled differentiation from other faces. This may not be an optimal solution but rather work in progress, since CAD parts with complex shapes might not unambiguously be characterized with this method.

Table 1: Characterization of parts and CAD surfaces

| **Part type** | **Position** | **Face count** | **Face ID** | **detection** | **Face type** | **Face direction** | **Face char type** | **Face char value** | **Area range min**  **[mm^2^]** | **Area range max**  **[mm^2^]** |
| --- | --- | --- | --- | --- | --- | --- | --- | --- | --- | --- |
| Frame_main_short | left | 2 | 1 | explicit | planar | x | area | max | 5,000 | 1,000,000 |
|  | “ | “ | 2 | “ | freeform | any | min_y | min | “ | “ |
|  | right | “ | 1 | “ | planar | x | area | max | “ | “ |
|  | “ | “ | 2 | “ | freeform | any | max_y | min | “ | “ |
| Frame_main_long | left | “ | 1 | “ | planar | x | area | max | “ | “ |
|  | “ | “ | 2 | “ | freeform | any | “ | max | “ | “ |
|  | right | “ | 1 | “ | planar | x | “ | max | “ | “ |
|  | “ | “ | 2 | “ | freeform | any | “ | min | “ | “ |
| Frame_door_back | left | “ | 1 | “ | planar | x | “ | max | “ | “ |
|  | “ | “ | 2 | “ | freeform | any | min_y | max | “ | “ |
|  | right | “ | 1 | “ | planar | x | area | max | “ | “ |
|  | “ | “ | 2 | “ | freeform | any | max_y | min | “ | “ |
| Stringer_short | all | 5 | 1 | implicit | planar | inwards | - | - | “ | “ |
|  | “ | “ | 2 | “ | “ | “ | - | - | “ | “ |
|  | “ | “ | 3 | “ | “ | “ | - | - | “ | “ |
|  | “ | “ | 4 | “ | “ | “ | - | - | “ | “ |
|  | “ | “ | 5 | “ | “ | “ | - | - | “ | “ |
| Stringer_long | “ | “ | 1 | “ | “ | “ | - | - | 30,000 | “ |
|  | “ | “ | 2 | “ | “ | “ | - | - | “ | “ |
|  | “ | “ | 3 | “ | “ | “ | - | - | “ | “ |
|  | “ | “ | 4 | “ | “ | “ | - | - | “ | “ |
|  | “ | “ | 5 | “ | “ | “ | - | - | “ | “ |

*Errors and occurrences during main process*

As outlined in our corresponding paper the repositioning procedure may fail for some CAD parts. Because the user needs to be given at hand some hints about why the algorithm aborted, there are several error messages that will be displayed in post process. The following Table 2 lists and explains the eventual errors. Regarding the occurrences, we refer to the corresponding paper, section *3.2. Back-End: Main Process*, steps 1 to 8.

Table 2: Error messages and occurrences in back-end process

|  | ***Error message*** | ***Explanation*** | ***Occurrence*** |
| --- | --- | --- | --- |
| 1 | “no or not enough CAD surfaces detected” | The imported part either does not match any given part characterization or it is categorized as part type but the CAD surfaces cannot be found with the given characterization | Step 3: Segmentation of CAD data |
| 2 | “no data in PCF1” or “no data in PCF2” | The abbreviations refer to “point cloud filter”. The partitioning of the scan failed, because there were no or not enough scan data in the surroundings of the CAD part. This may indicate that the scan data is either not captured properly or other components hide the corresponding regions. In addition, the CAD part may be in false position globally. | Step 4: Partitioning the scan |
| 3 | “no data in one or more PCF2 for faces” | Same as 2, but done for separate CAD faces | Step 4: Partitioning the scan |
| 4 | “no or not enough segments per face detected” | This error refers to failure of the abovementioned customized segmentation algorithm. The segmentation does not yield sufficient clusters in the scan data, s.t. a repositioning could be carried out properly. | Step 5: Segmentation of scan sections |
| 5 | “no data in one or more upsamled CAD faces”  “no data in one or more upsamled CAD faces ICP 1”  “no data in transformed CAD faces ICP 2” | In the context of getting rid of outliers and noise artefacts in the scan segments, upsampling or further outlier removal fails for the CAD part. This may also indicate that not enough scan data is present for corresponding CAD surfaces. | Step 6: Cleaning up segmentation results |
| 6 | “no or not enough reduced segments ICP 1 detected”  “no or not enough reduced segments ICP 2 detected” | Same as 5, but with regards to the scan section data. Outlier removal fails, or too many points are removed s.t. a later repositioning is not possible. | Step 6: Cleaning up segmentation results |
| 7 | “registration custom icp: has failed” | The custom ICP fails. This may indicate that the initial position of CAD compared to the scan data is too far or too distorted to the scan data. Also, it may occur that there are that little points left after clean up, that the CICP is not able to optimize the position in a proper way. Normally, the fault of too less points should be intercepted with errors 4-6. | Step 7: Repositioning the components |

*Exemplary result table for successful and failed repositioning*

The following tables Table 3 -Table 7 contain the results of the repositioning process. The columns are split into the five tables. The six rows show exemplary results of two door frames, two main frames and two stringers. Table 3 contains the columns part name, general type, type specification, number of computations and ICP type. The specifications correspond to Table 1 and are determined during computation. The number of computations may be larger than one, since it was a requirement to only translate and but also translate and rotate certain parts, yielding two new positions for a single part. Also in the computation count, it is outlined if the computation failed. If so, in the column ICP type, the error message is stored to be given the user at hand in the post process. In Table 4, the quantitative results begin. Translations (in [mm]) and rotations (in [rad]) in x, y, and z directions and further a displacement in the so-called “Top-Of-Seat-Track” (ToS) position. Table 5 continues with fitness-score values, also their maximum and minimum, divided in initial and final states. Finally, in columns of Table 6 and Table 7 the $4x4$ transformation matrix is stored.

Table 3: Exemplary computation results - columns 1-5

| **Name** | **Part type general** | **Part type specification** | **Computation count** | **ICP type** |
| --- | --- | --- | --- | --- |
| SEC-43_DOOR_FRAME-AFT_RH-1 | Frame | frame_door_front | 1 | Rotation+Translation |
| SEC-43_DOOR_FRAME-AFT_RH-1 | Frame | frame_door_front | 2 | Rotation |
| SEC_43_FRAME_777_IF-2 | Frame | frame_main_top | FAILED - NO RESULTS | Error - compute_segmentation_faces: no or not enough segments per face detected |
| SEC_43_FRAME_777_OF-2 | Frame | frame_main_top | FAILED - NO RESULTS | faces could not be determined |
| SEC_43_STRINGER_LH_11-1 | Stringer | stringer_long | 1 | Translation |
| SEC_43_STRINGER_LH_14-1 | Stringer | stringer_long | 1 | Translation |

Table 4: Exemplary computation results - columns 6-14

| **Transl x** | **Transl y** | **Transl z** | **Rot x** | **Rot y** | **Rot z** | **TOS delta x** | **TOS delta y** | **TOS delta z** |
| --- | --- | --- | --- | --- | --- | --- | --- | --- |
| 8.470001 | 0 | 0 | 0 | -0.158 | -0.02 | 8.470703 | 0 | 0 |
| 0 | 0 | 0 | 0 | 0.279 | -0.82 | 0 | 0 | 0 |
| - | - | - | - | - | - | - | - | - |
| - | - | - | - | - | - | - | - | - |
| 0 | 1.55 | 0.36 | 0 | 0 | 0 | 0 | 1.550049 | 0.359863 |
| 0 | 6.98 | -6.01 | 0 | 0 | 0 | 0 | 6.97998 | -6.009766 |

Table 5: Exemplary computation results - columns 15-28

| **Dist average initial** | **Dist average final** | **Dist min initial** | **Dist min final** | **Dist max initial** | **Dist max final** | **Dist min final x** | **Dist min final y** | **Dist min final z** | **Dist max final x** | **Dist max final y** | **Dist max final**  **z** | **Dist cad min** | **Dist cad max** |
| --- | --- | --- | --- | --- | --- | --- | --- | --- | --- | --- | --- | --- | --- |
| 3.34792 | 0.706475 | 0.009064 | 0 | 7.493674 | 3.516443 | 0 | 0 | 0 | 3.515625 | -0.001221 | 0.009766 | 1.645898 | 10.508287 |
| 3.34792 | 1.326086 | 0.009064 | 0.001539 | 7.493674 | 5.551144 | 0.001953 | 0 | 0 | 5.550781 | -0.079346 | -0.026855 | 0.233531 | 7.402332 |
| - | - | - | - | - | - | - | - | - | - | - | - | - | - |
| - | - | - | - | - | - | - | - | - | - | - | - | - | - |
| 1.004064 | 0.465338 | 0 | 0 | 3.428309 | 2.421124 | 0 | 0 | 0 | 0.011719 | -0.54248 | 2.359375 | 1.591274 | 1.591274 |
| 5.850525 | 2.033753 | 0.677917 | 0 | 12.509967 | 7.5293 | 0 | 0 | 0 | 0 | -5.549561 | 5.087891 | 9.210723 | 9.210723 |

Table 6: Exemplary computation results - columns 29-36

| **[1,1]** | **[2,1]** | **[3,1]** | **[4,1]** | **[1,2]** | **[2,2]** | **[3,2]** | **[4,2]** |
| --- | --- | --- | --- | --- | --- | --- | --- |
| 0.999996 | -0.000349 | 0.002758 | 0 | 0.000349 | 1 | 0 | 0 |
| 0.999886 | -0.014311 | -0.004869 | 0 | 0.014311 | 0.999898 | 0 | 0 |
| - | - | - | - | - | - | - | - |
| - | - | - | - | - | - | - | - |
| 1 | 0 | 0 | 0 | 0 | 1 | 0 | 0 |
| 1 | 0 | 0 | 0 | 0 | 1 | 0 | 0 |

Table 7: Exemplary computation results - columns 37-44

| **[1,3]** | **[2,3]** | **[3,3]** | **[4,3]** | **[1,4]** | **[2,4]** | **[3,4]** | **[4,4]** |
| --- | --- | --- | --- | --- | --- | --- | --- |
| -0.002758 | 0.000001 | 0.999996 | 0 | 21.570313 | 6.054932 | -47.852539 | 1 |
| 0.004869 | -0.00007 | 0.999988 | 0 | -62.480469 | 249.073975 | 84.592773 | 1 |
| - | - | - | - | - | - | - | - |
| - | - | - | - | - | - | - | - |
| 0 | 0 | 1 | 0 | 0 | 1.550049 | 0.359863 | 1 |
| 0 | 0 | 1 | 0 | 0 | 6.97998 | -6.00976 | 1 |
